# Sistema de alerta temprana para altas temperaturas y olas de calor: la necesidad de una política de salud pública en Colombia

**DOI:** 10.7705/biomedica.7934

**Published:** 2025-11-27

**Authors:** Alexander Salazar-Ceballos, Lídice Álvarez-Miño

**Affiliations:** 1 Grupo de Investigación GRISAL, Programa de Enfermería, Universidad Cooperativa de Colombia, Santa Marta, Colombia Universidad Cooperativa de Colombia Universidad Cooperativa de Colombia Santa Marta Colombia; 2 Grupo de Investigación GICCE, Programa de Enfermería, Universidad del Magdalena, Santa Marta, Colombia Universidad del Magdalena Universidad del Magdalena Santa Marta Colombia

**Keywords:** cambio climático, alerta temprana, calor extremo, poblaciones vulnerables, adulto mayor, mujer embarazada, niño, Climate change, early warning, extreme heat, vulnerable populations, aged, pregnant people, child

## Abstract

El aumento de la temperatura global, acelerado por el actual cambio climático, requiere la promoción de políticas de salud pública, como la generación de sistemas de alerta para altas temperaturas y olas de calor, para disminuir el riesgo de los grupos poblacionales más vulnerables: niños, mujeres gestantes y adultos mayores.

La evidencia científica compilada en esta revisión descriptiva de carácter narrativo respalda que las mujeres en estado de embarazo tienen un mayor riesgo de parto prematuro cuando han estado expuestas a altas temperaturas; los adultos y los niños pueden tener riesgo de deshidratación, y los adultos mayores de 65 años pueden tener un mayor riesgo de padecer enfermedades cardíacas.

Se analizaron algunos de los sistemas de alerta temprana de diferentes países para altas temperaturas y olas de calor, considerando un enfoque de disminución del riesgo para la salud de las personas.

En Colombia, la normatividad vigente permite la generación de un sistema de alerta temprana para las altas temperaturas. El *Plan decenal de salud pública 2022-2031*, consignado en la resolución 2367 de 2023, mediante el eje estratégico 5: “cambio climático, emergencias, desastres y pandemias”, invita a desarrollar un trabajo intersectorial para la gestión en salud asociada con estos fenómenos. Este plan tiene como meta estratégica territorial para el 2031 que los diferentes territorios generen sistemas de alerta temprana para disminuir el impacto de las amenazas exacerbadas por el cambio climático.

Finalmente, se presenta un marco de referencia basado en el riesgo climático y se invita a las instituciones gubernamentales a liderar este proceso.

## Abstract (en)

The rise in global temperatures, accelerated by ongoing climate change, requires promotion of public health policies, such as the creation of early warning systems for high temperatures and heatwaves, to reduce the risk to the most vulnerable population groups, including children, pregnant women, and older people.

Scientific evidence supports that pregnant women are at greater risk of preterm birth during exposure to high temperatures; adults and children may be at risk of dehydration, and adults over 65 years may be at higher risk of heart disease.

This paper analyzes some of the early warning systems for high temperatures and heatwaves in various countries, with a focus on reducing risks to human health. In Colombia, current regulations allow the creation of early warning systems for high temperatures. The *Plan decenal de salud pública 2022-2031*, established by Resolution 2367 of 2023, through strategic axis 5: “climate change, emergencies, disasters, and pandemics”, calls for the development of intersectoral work for health management associated with these phenomena. This plan has as its strategic territorial goal that the different territories generate early warning systems by 2031 to reduce the impact of the threats exacerbated by climate change.

Finally, we present a climate-risk-based framework and invite government institutions to lead this process.

El cambio climático es actualmente una de las amenazas más grandes para la salud pública y los grupos poblacionales más vulnerables recibirán el mayor impacto; por ello, es necesario generar y promover políticas de protección con enfoque diferencial desde la salud pública.

Entre las amenazas exacerbadas por el cambio climático, se encuentran las altas temperaturas y las olas de calor. Para enfrentar estas amenazas climáticas, se han implementado sistemas de alerta temprana, pero en Colombia, a la fecha, no se cuenta con este tipo de alertas. Por lo anterior, este documento pretende promover la necesidad de establecer un sistema de alerta temprana para altas temperaturas y olas de calor -con énfasis en la protección de la salud- para Colombia, como política de salud pública a partir del análisis reflexivo de la evidencia científica ^1^^,^^2^.

Se hizo una revisión descriptiva, narrativa y no sistemática ^3^, a partir de una búsqueda de artículos en Pubmed sobre el aumento de la temperatura y las olas de calor, su definición e impactos en la salud, con énfasis en la mortalidad, las poblaciones vulnerables y los sistemas de alerta temprana a nivel global. Además, se revisaron documentos institucionales -como marcos normativos- para proponer las características generales que debería tener un sistema de alertas tempranas.

## La amenaza del cambio climático para la salud

El cambio climático ha acelerado el aumento de la temperatura global, lo que, a su vez, se asocia con una mayor frecuencia e intensidad de las olas de calor y las altas temperaturas ^4^. El *Sixth Assessment Report of the Intergovernmental Panel* ha evidenciado que un aumento en la temperatura global de 0,5 °C tiene un impacto regional estadísticamente significativo ^5^. El 2024 fue el año más caliente registrado desde 1850, 0,72 °C más caliente que el promedio entre 1991 y el 2020 ^6^.

La exposición a las altas temperaturas conlleva diversos riesgos sobre la salud ^7^. Se ha estimado que cerca de un tercio de las muertes relacionadas con el calor se pueden atribuir al cambio climático. En Europa, el verano del 2022 fue considerado como el más caluroso reportado. Las temperaturas tuvieron máximos históricos según los reportes de los diferentes sistemas de monitoreo. Durante ese verano, se estimaron en cerca de 61.000 las muertes relacionadas con el calor, registradas en 35 países que representan un total de 543 millones de personas ^3^^,^^8^.

En un estudio reciente, se concluyó que las muertes relacionadas con las altas temperaturas eran un evento raro, pero dado el aumento continuo de la temperatura global, se prevé que las muertes por esta causa serán cada vez más frecuentes. En dicho estudio, se incluyeron diferentes países de varios continentes, entre ellos Colombia ^9^.

### Olas de calor en Latinoamérica según el informe *Lancet Countdown Latin America*

El reciente informe de *Lancet Countdown Latin America*^2^ presenta 34 indicadores para monitorear la relación entre el cambio climático y la salud. Estos indicadores constituyen una base científica para la toma de decisiones orientadas a mejorar la salud y el bienestar de las personas, y a reducir las inequidades sociales en Latinoamérica mediante acciones sobre el clima y la salud.

Dicho informe evidencia que en estos países también ha aumentado la exposición de los grupos poblacionales vulnerables a eventos climáticos extremos, como el de los niños y los mayores de 65 años a las olas de calor. En Latinoamérica, se observó un incremento cercano al 140 % en la mortalidad relacionada con el calor al comparar los periodos del 2000 al 2009 y del 2013 al 2022. Respecto a políticas de adaptación, las estrategias de los países latinoamericanos aún son débiles y presentan un grado limitado de integración entre los servicios meteorológicos y el sector salud ^2^.

## Mortalidad relacionada con las altas temperaturas en Colombia

En Colombia, la mortalidad relacionada con el calor extremo ha sido documentada en pocos artículos científicos. Lüthi *et al*. analizaron las muertes atribuibles a altas temperaturas en 748 localidades de 47 países, incluyendo cinco ciudades colombianas: Barranquilla, Bogotá, Cali, Cartagena y Medellín. En este estudio, se reportaron 957 muertes relacionadas con altas temperaturas entre el 1998 y el 2013, consideradas como un evento raro. Sin embargo, dado el aumento continuo de la temperatura global y según lo evidenciado por los modelos de frecuencia e impacto, los autores advierten que las muertes por esta causa serán cada vez más frecuentes ^9^.

Por su parte, Vicedo-Cabrera *et al*. estimaron la carga de mortalidad asociada con la exposición adicional al calor -como consecuencia del cambio climático inducido por el hombre- a partir del análisis de datos de 732 localidades en 43 países. Para Colombia, los investigadores reportaron 322.750 muertes asociadas con el calor entre 1998 y el 2013, y una temperatura media diaria de 23,9 (RIC: 23,1 a 24,6) para las cinco ciudades colombianas evaluadas ^10^.

En un reporte breve, Borchers-Arriagada *et al*. informaron sobre la mortalidad relacionada con temperaturas extremas entre el 2010 y el 2019 en los 32 departamentos de Colombia. Los autores encontraron que el 1,8 % de todas las muertes en Colombia se puede atribuir a temperaturas subóptimas (IC _95 %_: 1,3 a 2,25). Sin embargo, el riesgo asociado con el calor fue diez veces mayor que con el frío. Se evidenció que algunos departamentos tenían mayor riesgo que otros, por lo que se resalta la necesidad de crear estrategias de adaptación al cambio climático según los territorios ^11^.

## Grupos poblacionales más vulnerables ante las altas temperaturas

La evidencia científica ha resaltado la necesidad de proteger a los grupos poblacionales más vulnerables ante las altas temperaturas, entre ellos, los niños, las mujeres gestantes y los adultos mayores ^12^. Los adultos y los niños pueden tener riesgo de deshidratación; sin embargo, en los adultos mayores se ha documentado un riesgo mayor de desarrollar enfermedades cardiovasculares, entre otras ^13^. En los últimos años, se ha observado un aumento casi del 50 % del riesgo de mortalidad relacionado con el calor en adultos mayores de 65 años ^14^^,^^15^, debido a la mayor prevalencia de enfermedades crónicas, que elevan el riesgo de morbilidad y mortalidad durante los eventos de altas temperaturas ^16^.

Las mujeres en estado de embarazo son el grupo poblacional más susceptible, ya que la exposición al calor se ha asociado con un mayor riesgo de parto prematuro. Se ha encontrado que el aumento de un grado Celsius (1 °C) está correlacionado con la probabilidad de riesgo de parto prematuro en un 5 %, mientras que las mujeres gestantes expuestas a las olas de calor aumentan dicha probabilidad en un 16%. Además, la exposición a las altas temperaturas genera efectos nocivos durante la gestación, lo que afecta el crecimiento fetal. Según lo anterior, las mujeres gestantes son un grupo poblacional prioritario que debe ser considerado en la formulación de las políticas de salud pública relacionadas con la gestión del riesgo asociado con temperaturas extremas ^17^^,^^18^.

Igualmente, se ha observado la relación entre los eventos de altas temperaturas y el aumento de admisiones a salas de urgencias por causas relacionadas con la salud mental ^19^^,^^20^. Las mujeres con bajo nivel socioeconómico, escaso apoyo social y antecedentes de salud mental, también constituyen un grupo vulnerable ante la exposición a las altas temperaturas ^21^.

## Definición de ola de calor

No existe una definición universal de ola de calor, sin embargo, según la *World Meteorological Organization*, pueden ser períodos de altas temperaturas con una duración entre dos y tres días ^22^. Existen algunas precisiones sobre la definición de ola de calor según diversos autores. Una definición de ola de calor y altas temperaturas se puede basar en las temperaturas mínimas y máximas durante uno a tres días y entre los percentiles 90 y 97,5 ^23^^,^^24^.

Estas temperaturas dependen de la región afectada por la amenaza climática, por ejemplo, un estudio en San Diego (California) generó las alertas de olas de calor por subregiones ^23^.

Un evento de alta temperatura también se puede reconocer cuando el índice de calor máximo supera determinada temperatura (por ejemplo, 32 ^0^C) y alcanza un percentil máximo (99) en un período de referencia determinado y en una región específica ^25^.

## Sistemas de alerta temprana para calor

¿Qué es un sistema de alerta temprana por calor? Según el manual guía de la Organización Mundial de la Salud (OMS), desarrollado en conjunto con la *World Meteorological Organization*, un sistema de alerta temprana por calor está diseñado para advertir a los tomadores de decisiones y al público en general sobre la exposición a altas temperaturas, y brindar recomendaciones para disminuir sus efectos negativos sobre la salud ^22^.

La guía de la OMS presenta un marco para el desarrollo de sistemas de alerta temprana, el cual comienza con la definición de los límites de las temperaturas altas. Para ello, se establecen los indicadores relevantes -como las temperaturas media, máxima y mínima o el índice de calor- junto con la duración del evento en días. Una vez excedidos los límites establecidos, se generan las respectivas alertas (nivel bajo o alto) y se asignan los responsables de la activación y gestión del sistema ^22^.

Se ha observado que existen sistemas de alerta temprana basados solamente en la medición de la temperatura y en su duración. No obstante, varios investigadores han planteado la necesidad de complementar estos enfoques tradicionales con sistemas centrados en la protección de la salud de las poblaciones expuestas mediante una evaluación eficiente de los riesgos para la salud ^7^^,^^26^.

En la literatura se reporta que algunos países, regiones y ciudades han complementado sus sistemas de alerta temprana con la evaluación de los riesgos para la salud. Este es el caso de los modelos de Francia, Reino Unido, España y la ciudad de Filadelfia en los Estados Unidos ^7^.

## *Early Warnings for All:* el movimiento por la generación de sistemas de alerta temprana

Ante la amenaza del cambio climático, se espera el aumento de los eventos extremos, entre ellos, las altas temperaturas y las olas de calor. El movimiento *Early Warnings for All* impulsa el desarrollo de sistemas de alerta temprana para la protección de las poblaciones ante eventos ambientales y climáticos. Esta iniciativa tiene el respaldo de la *World Meteorological Organization* y de las Naciones Unidas para la Reducción del Riesgo de los Desastres ^27^. Una encuesta de la Organización de las Naciones Unidas evidenció que solo el 33 % de los 88 países que la respondieron manifestaron estar desarrollando o haber desarrollado sistemas de alerta temprana que integren la relación entre el calor y la salud ^28^.

## Sistemas de alerta temprana ante calor extremo con énfasis en la evaluación y la disminución del riesgo para la salud de las personas

Según un trabajo reciente, la mayoría de los modelos actuales de sistemas de alerta temprana no se han desarrollado adecuadamente con un modelo de riesgo salud-enfermedad. Algunos países han desarrollado sistemas basados en métodos tradicionales de monitoreo de la temperatura, combinados con la predicción de la duración de las olas de calor y la categorización de los niveles de alerta mediante un esquema de colores: rojo, amarillo y verde. Entre estos sistemas, se destacan los de Australia, China e India. Otros países, como Hungría y Sudáfrica, han implementado sistemas de alerta temprana basados únicamente en la temperatura ^7^.

Sin embargo, este enfoque no es sólido (*robust*) y depende de la variabilidad geográfica, particularmente relevante en países como Colombia, que presenta una amplia diversidad regional. Además, usualmente, estos sistemas no integran la evaluación del riesgo sobre la salud. Solo unos pocos países han desarrollado sistemas con énfasis en la evaluación y la disminución del riesgo para la salud de las personas, entre ellos, Reino Unido, Bélgica, Japón, México, Francia, España y la ciudad de Filadelfia ^7^^,^^29^. En el cuadro 1, se presenta la característica principal y el enlace de la plataforma oficial de cada sistema.

**Cuadro 1:** Análisis de diversos sistemas de alerta temprana ante las olas de calor, con un enfoque de evaluación del riesgo sobre la salud de las personas

País o ciudad	Nombre del sistema de alerta temprana	Característica central	Enlace
Reino Unido	*Heat Mortality Monitoring Report*	Enfoque de monitoreo y prevención de riesgos a partir de datos estructurados	https://www.gov.uk/government/publications/heat-mortality-monitoringreports/heat-mortality-monitoring-report-2023
Bélgica	Be-MOMO: *Belgian Mortality Monitoring*	Monitoreo en tiempo real para la toma rápida de decisiones en salud	https://epidata.sciensano.be/epistat/dashboard/#momo
Japón	*Heat Illness Prevention Information: Heat Stroke Alert*	Información preventiva y específica sobre el calor, con datos visuales	https://www.wbgt.env.go.jp/en/alert.php#alert_map_area
México	Vigilancia Epidemiológica de Temperaturas Naturales Extremas 2025	Genera recomendaciones en salud pública a partir de datos estructurados	https://www.gob.mx/salud/documentos/informes-semanales-para-la-vigilancia-epidemiologica-de-temperaturas-naturales-extremas-2025
Francia	*Vigilance Meteo France Canicule*	Orienta las acciones ante olas de calor con datos visuales	https://vigilance.meteofrance.fr/fr/canicule/demain
España	Riesgo Meteosalud	Consejos de salud a partir de información meteorológica integrada	https://www.aemet.es/es/portada
Filadelfia (USA)	*Hot Weather Preparedness*	Enfoque en acciones prácticas para los ciudadanos	https://www.phila.gov/es/departments/oem/ready-or-not/how-to-get-ready/natural-hazards/hot-weather-preparedness

El Reino Unido cuenta con el sistema *Heat Mortality Monitoring Report*, a cargo de la *UK Health and Security Agency*^30^. Desde el 2020, la plataforma genera un informe anual sobre la mortalidad asociada con las olas de calor. En el verano del 2023, se estimaron en 2.295 las muertes asociadas con cinco episodios de olas de calor, cada uno establecido por fechas específicas. Los informes incluyen análisis estadísticos de la mortalidad por cada ola de calor, además de datos por región, sexo y temperatura.

En Bélgica, el sistema de alerta es Be-MOMO (*Belgian Mortality Monitoring*), una plataforma en línea que, en un panel interactivo (*dashboard*), presenta un informe gráfico de la mortalidad por causa desde el 2020, por lo que permite visualizar la correlación entre la mortalidad y las temperaturas extremas. Esta plataforma está a cargo del Servicio de Epidemiología de Enfermedades Infecciosas de Sciensano ^31^.

Japón cuenta con el sistema *Heat Stroke Alert*, una plataforma en línea que proporciona información predictiva y emite alertas relacionadas con las altas temperaturas en las diferentes regiones del país. Su objetivo principal es advertir a las personas sobre los riesgos del calor extremo, fomentar la adopción de medidas preventivas y disminuir los efectos adversos sobre la salud. Esta plataforma es liderada por el Ministerio del Medio Ambiente y la Agencia Meteorológica de Japón ^32^.

Entre los países latinoamericanos, México ha desarrollado un sistema de alerta temprana para las olas de calor que incluye la evaluación de sus efectos sobre la salud. Este sistema genera un informe por semana epidemiológica. En México, la temporada de calor comprende las semanas epidemiológicas 12 a 40. El informe presenta el total acumulado de casos por semana y por región, e incluye los efectos sobre la salud asociados con las altas temperaturas, como deshidratación, golpes de calor, quemaduras y defunciones. Además, presenta una descripción de las temperaturas máximas ilustradas en un mapa geográfico. Esta plataforma es liderada por la Dirección General de Epidemiología de la Secretaría de Salud de México ^33^.

El sistema de alerta temprana de Francia incluye un mapa de vigilancia con niveles de alerta codificados por colores para facilitar su interpretación por parte del público. El sistema emite pronósticos con al menos un día de anticipación sobre la eventual ocurrencia de olas de calor, informa sobre posibles consecuencias y proporciona consejos generales y de salud, particularmente, cuando las alertas son de color naranja o rojo ^34^.

Por su parte, España tiene una plataforma, administrada por la Agencia Estatal de Meteorología en coordinación con el Ministerio de Sanidad, que integra datos meteorológicos con información sobre riesgos para la salud relacionados con el calor extremo; permite que los ciudadanos busquen la información para su provincia y municipio ^35^.

Finalmente, el sistema de la ciudad de Filadelfia es reconocido por ser uno de los pioneros, ya que se originó en el verano de 1995. Este sistema se centra en brindar instrucciones y recomendaciones específicas para climas calurosos extremos, y se enfoca en proporcionar acciones prácticas para los ciudadanos ^36^.

## *Plan Decenal de Salud Pública* y normas de gestión del riesgo climático en Colombia

El *Plan Decenal de Salud Pública 2022-2031*, establecido por medio de la Resolución 2367 de 2023 ^37^, mediante el eje estratégico 5: “cambio climático, emergencias, desastres y pandemias”, invita a desarrollar un trabajo intersectorial para la gestión del riesgo del cambio climático. Este trabajo intersectorial es viable gracias a la Ley 1523 de 2012 -Política Nacional de Gestión del Riesgo y Desastres- ^38^ y a la Ley 1931 de 2018 -por la cual se establece la gestión del cambio climático- ^39^, las cuales buscan disminuir el impacto de las amenazas exacerbadas por el cambio climático, como las altas temperaturas y las olas de calor en los diferentes grupos poblacionales. En línea con estas disposiciones, la meta estratégica del plan decenal al 2031 es que los territorios generen sistemas de alerta temprana para mitigar los efectos del cambio climático y reducir el riesgo de morbilidad y mortalidad en los grupos poblacionales más vulnerables: los niños, las mujeres gestantes y los adultos mayores.

### Propuesta de un marco de referencia para Colombia de sistemas de alerta temprana de olas de calor para disminuir el riesgo sobre la salud

Con base en la evidencia analizada, para la generación de un sistema de alerta temprana en Colombia se sugiere un marco de referencia basado en la comprensión del riesgo climático ^40^^,^^41^. El riesgo climático se define como la probabilidad de que los eventos climáticos extremos generen un impacto adverso sobre las personas, los ecosistemas y los bienes materiales. Este riesgo contempla tres dimensiones: 1) la amenaza climática (las olas de calor, las altas temperaturas), 2) la vulnerabilidad de los grupos poblacionales, y 3) la exposición, entendida como el nivel o grado de contacto con la amenaza (la presencia de grupos poblacionales vulnerables en zonas susceptibles al calor extremo).

Según la revisión hecha, los sistemas de alerta temprana deben integrar la información relacionada con la temperatura y contemplar acciones para disminuir el riesgo de exposición en los grupos poblacionales más vulnerables, como los niños, las mujeres embarazadas y los adultos mayores ^12^^,^^16^^,^^42^^-^^44^. Para el desarrollo de este sistema, se propone articular una colaboración entre el Sistema de Vigilancia en Salud, el Ministerio de Salud y Protección Social y el Instituto de Hidrología, Meteorología y Estudios Ambientales del Ministerio de Ambiente y Desarrollo Sostenible.

## Conclusión

Se hizo una revisión de algunos de los sistemas de alerta temprana para el monitoreo de temperaturas altas y olas de calor, con el objetivo de promover su diseño e implementación en Colombia, desde un enfoque preventivo del impacto de estas amenazas climáticas sobre la salud. La evidencia analizada permite concluir que estos sistemas analizan la información sobre las temperaturas y su impacto en la salud de forma retrospectiva o predictiva. No todos los países han generado estos sistemas, pero existe una iniciativa global al respecto: *Early Warning for All*.

Según la evidencia científica, en los últimos años se ha observado una tendencia orientada a la protección de los grupos poblacionales más vulnerables a estos eventos climáticos.

En Colombia, los organismos públicos llamados a liderar estos sistemas son el Ministerio de Salud y Protección Social y el Ministerio de Ambiente y Desarrollo Sostenible, con el acompañamiento de la comunidad académica de las diferentes regiones geográficas. Asimismo, el *Plan Decenal de Salud Pública* establece como meta de salud pública la generación de sistemas de alerta temprana, por lo que se espera su pronta implementación para el seguimiento de las altas temperaturas y las olas de calor, enfocada en prevenir y disminuir su impacto en los grupos poblacionales más vulnerables.
